# Antioxidant, Angiotensin-Converting Enzyme Inhibitory Properties and Blood-Pressure-Lowering Effect of Rice Bran Protein Hydrolysates

**DOI:** 10.3390/foods9060812

**Published:** 2020-06-20

**Authors:** Inajara Beatriz Brose Piotrowicz, Marta Garcés-Rimón, Silvia Moreno-Fernández, Amaya Aleixandre, Myriam Salas-Mellado, Marta Miguel-Castro

**Affiliations:** 1 Laboratório de Tecnologia de Alimentos, Escola de Química e Alimentos, Universidade Federal do Rio Grande, Rio Grande 96.203-900, Brasil; inabbp@yahoo.com.br (I.B.B.P.); mysame@yahoo.com (M.S.-M.); 2 Departamento de Bioactividad y Análisis de Alimentos, Instituto de Investigación en Ciencia de Alimentación (CIAL, CSIC-UAM), 28049 Madrid, Spain; marta.garces@ufv.es (M.G.-R.); silvia.moreno@csic.es (S.M.-F.); 3 Grupo de Investigación en Biotecnología Alimentaria, Universidad Francisco de Vitoria, 28223 Madrid, Spain; 4 Departamento de Farmacología, Facultad de Medicina, Universidad Complutense de Madrid, 28040 Madrid, Spain; amaya@med.ucm.es

**Keywords:** defatted rice bran, antioxidant hydrolysates, antihypertensive activity, bioactive compounds, hypertensive rats

## Abstract

This research aimed to investigate the biological properties of different hydrolysates derived from industrial and laboratory defatted rice bran proteins. Industrial and laboratory defatted rice bran protein concentrates were hydrolyzed with alcalase or flavorzyme. The degree of hydrolysis (DH), oxygen radical absorbance capacity (ORAC), reducing power, total phenolic compounds (TPC), and angiotensin-converting enzyme (ACE) inhibitory activity, were determined in the hydrolysates and the molecular fractions lower than 3 kDa. Systolic blood pressure (SBP) was measured using the tail-cuff method before and after oral administration of 80 mg/kg of different rice bran protein hydrolysate (RBPH) fractions lower than 3 kDa in male spontaneously hypertensive rats (SHR) and normotensive Wistar–Kyoto (WKY) rats. The highest values of in vitro antioxidant activity and TPC were observed in RBPH with alcalase defatted by industry (RBPH2A), and, in all cases, these bioactivities were higher in the molecular fractions lower than 3 kDa. Once again, fractions lower than 3 kDa obtained with alcalase showed a potent ACE inhibitory activity (RBPH1A<3 and RBPH2A<3). The administration of RBPH1A<3 caused a significant decrease in the SBP in SHR, where the maximum decrease was reached at 8 h after administration. SBP in WKY rats was not modified after the administration of RBPH1A<3. These results suggest that the rice bran protein hydrolysates obtained from industry after treatment with alcalase could be an interesting source of bioactive peptides, with potential action on hypertension and other related pathologies.

## 1. Introduction

Cardiovascular diseases (CVDs) are considered the most important health problem in industrialized countries. These pathologies represent the main cause of death worldwide, with hypertension being one of the main risk factors for developing CVD [1]. Hypertension is normally treated with drugs, in particular with angiotensin-converting enzyme (ACE) inhibitors. ACE plays an important physiological role in the regulation of blood pressure. Angiotensin-I is transformed to angiotensin-II by the action of ACE, resulting in arterial constriction and blood pressure elevation [2]. Since synthetic ACE inhibitors frequently produce side effects, research on natural compounds with ACE inhibitory properties can be advisable for decreasing the high values of arterial blood pressure and to replace synthetic drugs [3]. In addition, there is mounting evidence linking oxidative stress with the development of a large number of diseases, including hypertension [4]. Antioxidant compounds with properties that inhibit cell oxidation could also be a valuable strategy to improve oxidative stress and related cardiovascular diseases.

In the last few years, the food industry has shown an increased interest in the study of bioactive compounds from natural sources as an alternative in the search for new therapeutic agents and treatments to improve human health. Among all the food components, proteins from animals and plants are considered one of the main sources to obtain functional ingredients. In this context, food-derived peptides are defined as inactive amino acid sequences within the protein but which carry out certain physiological functions in the body after its release by in vivo (gastrointestinal digestion) and in vitro (chemical or enzymatic) hydrolysis. They have demonstrated potential health benefits against several chronic diseases, in particular hypertension [3,5,6]. In fact, they could represent a good and safe strategy for controlling high blood pressure and some related cardiovascular diseases due to their high enzymatic affinity, strong specificity, and low toxicity [7]. Recently, plant-protein-derived hydrolysates, mainly from soybean, have received special attention to produce bioactive peptides [8,9].

Rice bran is an abundant byproduct in the rice milling industry, which is used to produce edible rice oil and animal feed [10,11]. Since rice bran is considered an important source of proteins (12.6–15.4%) [12], an interesting alternative may be the production of protein concentrates to obtain hydrolysates and peptides with bioactive properties [7,13]. Rice bran enzymatic hydrolysates and peptides have exerted various in vitro biological activities, including antioxidant, antidiabetic, anticancer, and ACE inhibition, but very little is known about the in vivo effects in animal models [14].

For all these reasons, the present study hypothesized that the production of different bioactive hydrolysates, derived from rice bran, might improve oxidative stress, hypertension, and/or other cardiovascular-related diseases.

The main aims of this work were: (1) to obtain hydrolysates from industrial and laboratory defatted rice bran protein concentrates using different food-grade microbial enzymes, (2) to investigate the in vitro antioxidant and ACE inhibitory properties of these hydrolysates and (3) to evaluate the in vivo activity of the selected rice bran protein hydrolysates in spontaneously hypertensive rats (SHR) and Wistar–Kyoto (WKY) rats.

## 2. Materials and Methods

### 2.1. Chemicals and Reagents

The enzymes alcalase 2.4L FG (E.C. 3.4.21.62, 2.4 AU/g), an endopeptidase from *Bacillus licheniformis* and flavorzyme 1000L (E. C. 232.752.2, 1000 U/g), a mixture of endo- and exo-peptidase from *Aspergillus oryzae* (both from Novozymes A/S, Bagsvaerd, Denmark), were kindly provided by LNF Latino Americana (Bento Gonçalves, Brazil). 2,2′-Azobis (2-metylpropionamidine) dihydrochloride (AAPH), Fluorescein, Trolox, and ACE (EC 3.4.15.1) were purchased from Sigma-Aldrich (St Louis, MO, USA). 2-Aminobenzoylglycyl-4-nitrophenylalanyl-proline (Abz-Gly-Phe (NO2)-Pro) was purchased from Bachem (Bunbendorf, Switzerland). Potassium ferricyanide was purchased from Dinamica (São Paulo, Brazil) and ferric chloride was purchased from Synth (São Paulo, Brazil). Captopril was provided by Sigma (Sigma-Aldrich Log., Schnelldorf, Germany).

### 2.2. Defatted Rice Bran Protein Concentrates

Long rice grain (*Orysa sativa* L.) was used in this study. The defatted rice bran protein concentrates were produced using two different processes; a concentrate obtained in the laboratory using a pH-shifting process under conditions previously described by Piotrowicz and Salas-Mellado [15] (DRBPC-1) and a concentrate provided by Irgovel and Josapar Industries (Pelotas, Brazil) (DRBPC-2). The degree of milling was 0.335 mm for the defatting process and 0.15 for the concentrating process. In the industrial process, the brown bran was submitted to a pelleting process (80 °C, 0.3 kg/cm^2^) before the lipid extraction, and the hexane evaporation process (90 °C, 1.5 kg/cm^2^) was performed after the extraction.

Proteins from DRBPC-1 and DRBPC-2 were solubilized at an alkaline pH (pH 11.0) using NaOH 1 M at 40 °C for 60 min, and the respective suspension was centrifuged at 8670× *g* for 20 min at 20 °C. The supernatant was collected and once again precipitated at pH 4.5 at 40 °C for 30 min and centrifuged under the same conditions described above. The precipitate obtained was suspended again in water, neutralized with a 1.0 M NaOH solution, and freeze-dried (LIDTDP L108, Liotop, São Carlos, Brazil) to obtain the dry concentrates DRBPC-1 and DRBPC-2.

The proximal compositions of DRBPC-1 and DRBPC-2 were 63.6% and 62.1% protein, 20.2% and 15.1% fat, 4.3% and 5.4% ash, and 11.9% and 17.4% carbohydrates (dry basis), respectively [14].

### 2.3. Preparation of Defatted Rice Bran Protein Hydrolysates

DRBPC-1 and DRBPC-2 concentrates were diluted at 2% (*w*/*v*) and hydrolyzed using 0.1 U/mg protein of alcalase or flavorzyme, respectively. The pH was adjusted to 8.0 for alcalase and to 7.0 for flavorzyme with a 1.0 N NaOH solution, according to the manufacturer’s specifications. Briefly, the hydrolysis process was carried out at 50 °C under constant stirring in a thermostatic water bath for 10 h to obtain peptide fragments that were small enough to resist the digestive process. The enzymatic hydrolysis was stopped via heating in a boiling water bath for 15 min (85 °C) and then centrifuged at 8700× *g* at 20 °C for 10 min. The supernatant was collected and freeze-dried to obtain two rice bran protein hydrolysates with alcalase (RBPH-1A and RBPH-2A) and two rice bran proteins hydrolysates with flavorzyme (RBPH-1F and RBPH-2F).

### 2.4. Ultrafiltration Process of Rice Bran Protein Hydrolysates

RBPH-1A, RBPH-2A, RBPH-1F, and RBPH-2F hydrolysates were diluted at 0.75% (*w*/*v*) and these solutions were submitted to an ultrafiltration process using a 3 kDa membrane (Millipore Corporation, Billerica, MA, USA) in a 300 mL ultrafiltration cell system (Amicon^®^ stirred cells, Merk Millipore, Madrid, Spain). Nitrogen gas pressure was used for pressurizing the cell and was applied directly to the ultrafiltration cell. The transmembrane pressure was kept constant at 60 psi. The ultrafiltration cell was placed on the magnetic stirring table, which was used to homogenate the RBPH solutions. Permeates were freeze-dried, obtaining one fraction of each hydrolysate lower than 3 kDa, which were named RBPH-1A<3, RBPH-2A<3, RBPH-1F<3, and RBPH-2F<3.

### 2.5. Degree of Hydrolysis Determination

The kinetics of the degree of hydrolysis (DH) of RBPH-1A, RBPH-1F, RBPH-2A, and RBPH-2F were determined through the pH-stat process, previously described by Adler-Nissen [16]. The quantification of DH using the pH-stat method was carried out by maintaining a constant pH value with standardized 0.2 N NaOH. The percentage of DH was calculated using the following equation:

DH = (h/h_total_) = ((B × N_B_)/(α × M_p_)) × (1/h_total_), (1)

where B is the base consumption (mL), N_B_ the normality of the base, α the average degree of dissociation of the α-NH_2_ groups, M_P_ is the mass of protein being hydrolyzed (g), and h_total_ the total number of peptide bonds in the protein substrate (meq/g protein).

The degree of dissociation (α) of the α-NH_2_ groups was calculated using the equation:

α = (10^pH − pK^)/(1 + 10^pH − pK^), (2)

where pK is the average dissociation value of the α-amino groups liberated during hydrolysis, which is dependent on the temperature, peptide chain length, and the nature of the terminal amino acid. At 50 °C (the hydrolysis temperature used in the present study), the average pK value of the α-amino groups of peptides and proteins is 7.1 [7]. At pHs 7.0 and 8.0, α was calculated to be 0.4 and 0.9 for flavorzyme and alcalase, respectively. The parameter h_total_ is given as meq peptide bonds per gram of protein, and for rice protein, this corresponds to 8.38 meq/g protein [16].

### 2.6. In Vitro Assays

#### 2.6.1. Oxygen Radical Absorbance Capacity (ORAC)

The antioxidant activity was determined in the defatted rice bran protein concentrates, their respective hydrolysates, and their respective fractions smaller than 3kDa using the ORAC assay, according to Garcés-Rimón et al. [17]. The scavenging activity of free radicals generated by the decomposition of 2,2′-azobis-(2-metylpropionamidine) dihydrochloride (AAPH) at 37 °C was evaluated. All reagents and samples were prepared in a 75 mM potassium phosphate buffer with pH 7.4. A black 96-well flat-bottom polystyrene microplate was used to transfer 20 µL of the different samples, standards, or buffer solution (blank), in triplicate, followed by adding 120 µL of 116.61 nM fluorescein solution. The mixture was incubated at 37 °C for 10 min in a microplate reader (FLUOstar OPTIMA, BMG Labtech, Offenburg, Germany), and then 25 µL of 14 mM AAPH was added. The fluorescence was recorded at 37 °C every minute for 90 min using a fluorimeter (FLUOstar OPTIMA, BMG Labtech, Offenburg, Germany), with excitation and emission wavelengths of 480 and 520 nm, respectively. All samples were tested in triplicate.

Trolox solutions (vitamin E analogue; 0.4–1.4 µM) were used as a standard curve. The net area under the curve (AUC) was calculated using:

AUC = 1 + ∑ ^i = 0–90^ (f_i_/f_0_), (3)

where f_0_ represents the initial fluorescence reading at 0 min and f_i_ represents the fluorescence reading at time “i” (range time of 0 to 90 min). The final ORAC values were calculated using the regression equation between the Trolox concentration and the net AUC, and were expressed as µmol eq Trolox/g sample. The net AUC of the samples were calculated as follows:

AUC = AUC _sample_ − AUC _blank_. (4)

#### 2.6.2. Reducing Power

The reducing power of the defatted rice bran protein concentrates, their respective hydrolysates, and their respective fractions smaller than 3kDa were measured according to the method described by Oyaizu [18] with some modifications. The reaction mixture containing 1.0 mL of sample solution (2.5 mg/mL) and 1.0 mL of phosphate buffer (0.2 mol/L, pH 6.6) was incubated with 1.0 mL of potassium ferricyanide (1% *w*/*v*) at 50 °C for 20 min. The reaction was terminated via the addition of trichloroacetic acid solution (1.0 mL, 10% *w*/*v*), and the mixture was centrifuged at 8760 *g* for 5 min. The upper layer of solution (1 mL) was mixed with distilled water (1 mL) and ferric chloride (0.2 mL; 0.1% *w*/*v*), and then the absorbance of the mixture was measured at 700 nm (BIOSPECTRO SP/22, São Paulo, Brazil) against a blank. An increasing absorbance of the reaction mixture indicates an increase in the reducing power of the sample.

#### 2.6.3. Total Phenolic Compounds Analysis

The total phenolic compounds (TPCs) were determined in the defatted rice bran protein concentrates, along with their respective hydrolysates and fractions smaller than 3kDa, by using the Folin–Ciocalteu assay with modifications. First, the different samples were homogenized with methanol (1 mg/mL) in an ultrasonic bath for 15 min. The suspensions were centrifuged at 5000 rpm for 10 min. An aliquot (40 µL) of the different extracts, standard solution of Gallic acid (30 to 500 µg/mL), or water (blank) were added to an Eppendorf tube of 2 mL, and 40 µL of Folin-Ciocalteu phenol reagent was added. After 5 min, 0.8 mL of 7% Na_2_CO_3_ solution was added to the mixture. The volume was made up to 2 mL with water. After incubation for 45 min at room temperature, 200 µL of colored suspension was added in a 96-well polystyrene microplate and the absorbance against the reagent blank was determined at 750 nm using a UV-visible spectrophotometer (FLUOstar OPTIMA, BMG Labtech, Offenburg, Germany). The total phenolic content was expressed as mg gallic acid equivalents (GAE)/g of the sample.

#### 2.6.4. Angiotensin-Converting Enzyme (ACE) Inhibitory Activity

ACE-inhibition was measured in the fractions smaller than 3 kDa—RBPH-1A<3, RBPH-2A<3, RBPH-1F<3, and RBPH-2F<3—according to Garcés-Rimón et al. [17]. The substrate o-aminobenzoylglycine-p-nitrophenylalanyl-proline (o-Abz-Gly-Phe (NO_2_)–Pro) was dissolved (0.45 mM) in a 0.15 M Tris buffer with 1.125 M NaCl at pH 8.3. ACE was prepared at a concentration of 0.04 U/mL of the enzyme in a 0.15 M Tris buffer containing 0.1 mM ZnCl_2_ at pH 8.3 (enzyme work solution). The reaction was performed by adding 40 µL of pure water or sample solution in a black 96-well flat-bottom polystyrene microplate, and each well was adjusted to 80 µL with a buffer solution or enzyme solution. The enzyme reaction was started by adding 160 µL of the substrate to the wells. The fluorescence generated was measured at 37 °C after 30 min using a fluorimeter (FLUOstar OPTIMA, BMG Labtech, Offenburg, Germany), with excitation and emission wavelengths of 350 and 420 nm, respectively. Triplicate tests were performed for each sample. The inhibitory activity was expressed as the concentration of protein required to inhibit 50% of the ACE activity (IC_50_), considering a coefficient of variation lower than 10% and correlation coefficient (R^2^) higher than 98%.

### 2.7. In Vivo Assays

#### 2.7.1. Animal Ethical Approval

All experiments were conducted in compliance with the guidelines for biomedical research stated by the European and Spanish legislation on the care and use of experimental animals (EU Directive 2010/63/EU for animal experiments; R.D. 53/2013). The experiments were approved by the Ethic Committee on Animal Use at Universidad Complutense de Madrid, Spain. The experiments were also designed to minimize the number of animals used and their suffering.

#### 2.7.2. Experimental Procedure in Spontaneously Hypertensive Rats

In this study, we used 17–24-week-old male spontaneously hypertensive rats (SHR), weighing 310–320 g, and 17–20-week-old male normotensive Wistar–Kyoto (WKY) rats, weighing 330–350 g. All these animals were obtained from Charles River Laboratories (Spain). During the treatment, the manipulation of the animals was performed following the appropriate safety measures and general health conditions. The animals were maintained at a temperature of 23 °C with 12-h light/dark cycles and consumed tap water and a standard diet for rats (A04 Panlab, Barcelona, Spain) *ad libitum* during the experimental period.

The different fractions smaller than 3 kDa—RBPH-1A<3, RBPH-2A<3, RBPH-1F<3, and RBPH-2F<3—were used at doses of 80 mg/kg, and orally administered by gastric intubation to the SHR in the morning between 8 and 9 am. Distilled water (negative control) and 50 mg/kg Captopril (positive control), a known antihypertensive drug, were also administered to the SHR using the same procedure. In these trials, the volume orally administered to the rats was always 1 mL/rat of either water or the appropriate treatment. Systolic blood pressure (SBP) was recorded in the rats using the tail-cuff method [19] before administration, and at 2, 4, 6, 8, 24, and 48 h post-administration. Before the measurement, the rats were kept at 38 °C for 10 min to detect the pulsations of the tail artery. To establish the SBP value, five reliable measurements were taken, and the average of all of them was obtained. To minimize stress-induced variations in blood pressure, all measurements were taken by the same person and in the same peaceful environment. Moreover, to guarantee the reliability of the measurements, a training period of two weeks before the actual trial time was established, and, during this period, the rats were accustomed to the procedure.

### 2.8. Statistical Analysis of In Vitro and In Vivo Assays

The results obtained from in vitro assays were expressed as the mean values ± SD (standard deviation) of three determinations. Statistical comparisons of the results were performed using one-way ANOVA and Tukey’s test. Values of *p* < 0.05 were considered significant, which were found using statistical software Statistica 7.0 (Statsoft, Sao Paulo, Brazil).

The results obtained from the in vivo assays were expressed as means ± SEM of the values obtained in the animals used in each experiment. The arterial blood pressure results were analyzed using two-way ANOVA with GraphPad Prism 6 Software (San Diego, CA, USA) for a minimum of eight rats, and differences between the groups were assessed using the Bonferroni test and were considered significant when *p* < 0.05.

## 3. Results and Discussion

**Figure 1:**
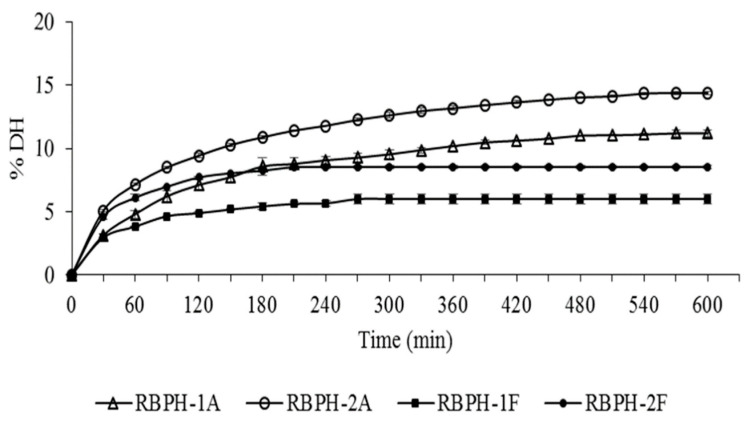
Degree of hydrolysis (%DH) of defatted rice bran protein concentrates RBPC-1 and RBPC-2 incubated with alcalase or flavorzyme enzymes. RBPH-1A: concentrate DRBPC-1 hydrolyzed with alcalase; RBPH-2A: concentrate DRBPC-2 hydrolyzed with alcalase; RBPH-1F: concentrate DRBPC-1 hydrolyzed with flavorzyme; RBPH-2F: concentrate DRBPC-2 hydrolyzed with flavorzyme. Each point of the curve shows the means ± standard deviation of three measurements.

Several methods are being used to produce food bioactive peptides, where the enzymatic hydrolysis process is one of the most used methods. Thus, the protein source, type of enzyme, and degree of hydrolysis (DH) define the characteristics and bioactivities of the peptides present in the hydrolysates [20]. DRBPC-1 and DRBPC-2 samples were subjected to a hydrolysis reaction with two food-grade microbial enzymes: alcalase or flavorzyme. Figure 1 shows the hydrolysis kinetics of the different defatted rice bran protein concentrates with the different enzymes for 10 h.

The DH increased with hydrolysis time, showing a gradual release of peptide fragments during the process. The curves present a high initial reaction rate followed by a decrease up to the stationary period, in which the degree of hydrolysis became constant. The stationary period was reached faster for the products obtained using flavorzyme, in 210 min (RBPH-2F) and 270 min (RBPH-1F) of hydrolysis. Hydrolysates using alcalase reached their stationary period after 480 min of reaction. At the end of the hydrolysis period, the DH was 10.4%, 13.4%, 4.2%, and 5.9% for RBPH-1A, RBPH-2A, RBPH-1F, and RBPH-2F, respectively. Thamnarathip, Jangchud, Jangchud, and Vardhanabhuti [21] also presented similar values of DH using rice bran hydrolyzed with alcalase (18.7%) compared to flavorzyme (7.4%) after 360 min of reaction. These results suggest that the specificity of the protease used to release peptides plays a very important role, independent of incubation time with enzymes. The action of the alcalase was more effective in both concentrates. However, both enzymes worked faster with the rice bran protein concentrates defatted by an industrial process (DRBPC-2) than with those obtained by our laboratory extraction process (DRBPC-1). This could be explained by the fact that, in some industries, the crude rice bran is submitted to a pelletization process to preserve the bran, where an agglomeration of milled particles occurs via mechanical processes combined with moisture, heat, and pressure conditions, to stabilize its lipolytic enzymes and to prevent lipid degradation, that could result on rancidity [22]. The industrial conditions can promote protein changes, modifying its structure produced via denaturation, and it is known that the best action of enzymes is obtained after protein denaturation [23]. Similar results were obtained by Uraipong and Zhao [7] after 4 h of hydrolysis of rice bran albumin using alcalase plus flavorzyme, and Sbroggio et al. observed the same behavior after denaturation using okara protein [24].

Despite both enzymes being from microbial origin, alcalase is an endopeptidase and has a limited range of specificity of peptide bonds for hydrolysis [25]. On the other hand, flavorzyme is a mixture of an endopeptidase and exopeptidase produced by *Aspergillus oryzae*, which can exert a broader range of action and therefore a higher DH would be expected with this enzyme [25]. Contrary to expectations, in our study, we obtained higher DH in alcalase hydrolysates.

As mentioned in the introduction section, enzymatic proteolysis may change food properties, such as digestibility, nutritional, and sensory quality, but provides health benefits due to the formation of bioactive peptides. The length and characteristics of the peptides formed, in terms of their constituent amino acids, the presence of polar and ionizable groups, and their hydrophobicity, determine the functional and bioactive properties of food hydrolysates; these depend not only on the degree of hydrolysis but also on the specificity of the enzyme and protein used as a substrate [26].

Natural antioxidants from food proteins have been investigated by several research groups because of their low price, high activity, and easy absorption [27]. Food hydrolysates with potential antioxidant activity are related to its composition, structure, hydrophobicity, and amino acid position in the peptide sequence [28], and these characteristics also seem to influence the antioxidant mechanism. Despite the mechanisms not being clear, different studies show that some peptides can act as inhibitors of lipid peroxidation [29], free radical neutralizers [29,30], and chelating agents of transition metallic ions [28]. Another important consequence of protein hydrolysis is the exposition of the active R-groups of the amino acids, and then the increase of its antioxidant power [30]. ORAC (ROO•) was recommended as a standard method by Prior, Wu, and Schaich [31] to be used as routine quality control and measurement of food antioxidant capacity. It is a HAT-based (hydrogen-atom-transfer-based) method that uses a controllable source of peroxyl radicals, where it can detect both hydrophilic and hydrophobic antioxidants by altering the radical sources and solvent. For that reason, ORAC (ROO•) is recommended for analyzing the antioxidant capacity of vegetables if only one method is justifiable [32]. Nevertheless, since there is not only one standardized official method, evaluating this property using different methods and conditions of the oxidation process is suggested [33]. Therefore, to evaluate the antioxidant capacity of rice bran proteins, the ORAC method and reducing power assays were performed (Table 1).

The antioxidant activity obtained using the ORAC method was higher in DRBPC-2 than in DRBPC-1, independent of the enzyme used. The high temperature and pressure used in the industry process might modify the protein structure to obtain different active products compared with native protein [10]. Ou et al. [34] studied the antioxidant activity of vegetable sources using the ORAC method and obtained values ranging from 18 µmol/g (for pea) to 160 µmol/g (for green pepper). These values were lower than those obtained from our RBPH.

The antioxidant activity significantly increased after hydrolysis with both food enzymes, and this activity increased by between two and three times in fractions smaller than 3 kDa, which showed the highest antioxidant activities. The ultrafiltration is usually used to enrich biologically food peptides and this process confirms that the antioxidant activity can be mainly attributable to low-molecular-mass peptides. Wattanasiritham et al. [10] worked with native and denatured protein fractions (albumin, globulin, and glutelin) that were non-hydrolyzed and hydrolyzed with pepsin and papain, and these researchers observed values from 100 µmol eq Trolox/g protein for native protein, 1500 µmol eq Trolox/g protein for hydrolyzed protein with papain, and 1000 µmol eq Trolox/g protein for hydrolysates with pepsin. In our study, a potent antioxidant activity was found using the ORAC method in fractions with a low molecular weight, with means ranging from 434.79 to 1916.15 µmol eq Trolox/g protein.

Reducing power, which measures the transformation of the Fe^3+^ of a ferricyanide complex to the ferrous form, was determined using the absorbance values of the samples, where a high absorbance is related to a high reducing power. This method argues that the ability to reduce iron has little relationship to the radical quenching processes (HAT) mediated by most antioxidants. However, the radical oxidation or reduction to the ions still stops the radical formation and the reducing power could reflect the ability of compounds to modulate the redox tonus in plasma and tissues [29]. When analyzing the results (Table 1), it was found that the products obtained from DRBPC-2 with alcalase after hydrolysis and ultrafiltration processes did not increase the reducing power. However, a significant increase in the absorbance values of the fractions smaller than 3 kDa obtained with flavorzyme was observed, suggesting an improvement in the reducing power. Zhang et al. [35] also obtained higher reducing power levels in fractionated protein hydrolysates from heat-stable defatted rice bran.

Rice is a rich source of many bioactive compounds, including special phenolic acids (such as ferulic acid, p-coumaric, and diferulate), which are not present in significant quantities in fruits or other vegetables [36]. These molecules exert antioxidant activity, where this property depends on the number and the position of hydroxyl groups on the phenolic ring [37]. Previous researchers have postulated the release of phenolic compounds after hydrolysis treatment. In fact, Chanput et al. [13] mentioned that the crude hordein fraction (a prolamin glycoprotein that presents in barley and some other cereals) showed the lowest phenolic content compared to their hydrolysates, and these researchers considered that this might be because of the unfolding of the protein, exposing the phenolic groups that were originally enclosed, suggesting that phenolic compounds could also be implicated in the high antioxidant activity observed after the hydrolysis of rice bran. To establish where our hydrolysis conditions allowed for releasing phenolic compounds and to know their participation in the antioxidant properties of the hydrolysate, we performed an analysis of the total phenolic compounds (Table 2).

Since both amino acids and phenolic compounds can show HAT ability in their molecular structure, the results obtained for the antioxidant capacity using ORAC could be linked not just with the hydrolysis process and ultrafiltration but also with the TPC released in these rice bran products. The positive relation between TPC and ORAC results, with a Pearson’s ratio coefficient of 0.98, could explain the antioxidant mechanisms underlying these products.

Table 3 shows the ACE inhibitory activity of the fractions smaller than 3 kDa obtained from the two types of DRBPC, hydrolyzed with either alcalase or flavorzyme, followed by the ultrafiltration process (RBPH-1A<3, RBPH-2A<3, RBPH-1F<3, RBPH-2F<3).

RBPH-1A<3 showed higher inhibitory activity than those hydrolyzed with flavorzyme. The IC_50_ value observed in RBPH-1A<3 was low (0.15 ± 0.02 mg/mL) and was within the range reported in the literature for other ACE inhibitory food hydrolysates with antihypertensive activity demonstrated in experimental models [38]. Regarding rice-derived hydrolysates, Chen et al. [39] worked with hydrolysates of rice prepared with alcalase and trypsin, where they obtained IC_50_ values of 0.28 mg/mL. These researchers, obtained fractions using macroporous resin, and the elution with ethanol 50% presented a lower IC_50_ value, reaching a value of 0.17 mg/mL, similar to that presented by the fraction RBPH-1A<3 [38]. Wang et al. [40] evaluated hydrolyzed from rice bran protein prepared with trypsin, showing IC_50_ = 0.30 mg/mL. These results suggest that an RBPH-1A<3 fraction could be a promising source of peptide sequences with antihypertensive properties.

Some physiological processes, such as changes in structure and function of the peptides during ingestion, digestion, and/or absorption, could be occurring, and the bioactive sequences could be destroyed by gastrointestinal proteases to produce inactive peptides or to become more biologically active. For this reason, in this study, we also conducted experiments on SHR to explore the in vivo antihypertensive activity of the hydrolysates of the industrial by-product of rice-milling.

Essential hypertension is one of the most common risk factors for the development of cardiovascular diseases [41]. SHR are known as a genetic model of hypertension, and, since its development in 1963 by Okamoto and Aoki, these animals are one of the most used experimental models for evaluating the antihypertensive properties of different compounds and to investigate the mechanism of action underlying their antihypertensive effect. In vivo effects are usually tested in this strain because the hypertension presented in this animal model is similar to the essential hypertension that occurs in humans [42,43,44]. Hypertension appears in both humans and SHR at an early age, the risk increases with a family history of hypertension, and the disease gets worse with the consumption of a sodium-rich diet [45]. This experimental model acquires arterial hypertension after five weeks old, presenting a level of pressure that is considered to be spontaneous hypertension between weeks 7 and 15. The plateau is reached between weeks 20 and 28, presenting no difference between the sexes [42].

**Figure 2:**
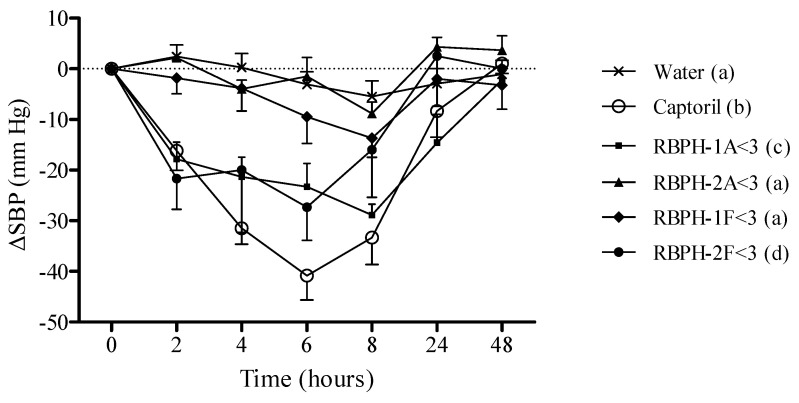
Decrease in systolic blood pressure (SBP) caused in spontaneously hypertensive rats (SHR) after the oral administration of water (x), Captopril (50 mg/kg) (○), or 80 mg/kg of the rice bran protein hydrolysate fractions with a molecular mass smaller than 3 kDa: RBPH-1A<3 (■), RBPH-2A<3 (▲), RBPH-1F<3 (♦), and RBPH-2F<3 (●). Data are expressed as means ± SEM. The experimental groups always had a minimum of eight animals. The same letters indicate no statistical differences (*p* > 0.05). Significance was estimated using two-way ANOVA.

The oral administration of distilled water to SHR in this study did not change the values of the SBP (Figure 2).

The greatest decreases in the SBP were obtained after the administration of 50 mg/kg of Captopril to the rats. The maximal decreases were observed 6 h after the administration of Captopril, and this variable returned to baseline 48 h after the administration (Figure 2). It was not surprising to find a clear decrease in the SBP when we administered 50 mg/kg of Captopril to the SHR because this drug is a potent antihypertensive and ACE-inhibitor with an IC_50_ value of 0.02 μM [46].

After a single oral administration of 80 mg/kg of the different RBPH fractions lower than 3 kDa, only RBPH-1A<3 and RBPH-2F<3 showed significant decreases of the SBP in SHR. The maximum decrease of the SBP values after administration of RBPH-1A<3 was reached 8 h after administration, and the SBP returned to baseline 24 h afterward (Figure 2). The maximum decrease caused by RBPH-2F<3 was observed 6 h after administration but the SBP returned to baseline 8 h afterward. The antihypertensive effect of RBPH-1A<3 and RBPH-2F<3 compared favorably with the blood-pressure-lowering effect of other food protein hydrolysates. The antihypertensive effect of the administration of 600 mg/kg rice bran protein hydrolysate with alcalase in SHR showed a maximum reduction of blood pressure (25 mm Hg) 6 h after oral administration [39]. In our study, we found a reduction of 30 mm Hg approximately 8 h after the oral administration of RBPH-1A<3 at much lower doses (80 mg/kg). In previous studies, we used different doses of egg white hydrolysate fractions with a 3 kDa cut-off membrane (25, 50, and 100 mg/kg) and we also found similar reductions (28 mm Hg) in the SBP in SHR [47]. Chen et al. [48] also evaluated different doses (1, 10, and 50 mg/kg) of the rice dreg protein hydrolyzed with trypsin. A decrease in the maximum blood pressure was observed 1 h after the oral administration of the 50 mg/kg dose (29 mm Hg), returning to baseline values after 7 h.

It is well known that blood pressure variability may contribute to organ damage [49]; therefore, the use of strategies with long-lasting antihypertensive effects is always desirable. In this context, it is important to highlight that the decrease in arterial blood pressure caused by RBPH-1A<3 lasted for a longer period than the antihypertensive effect observed with RBPH-2F<3. In accordance with this idea, the antihypertensive properties of RBPH-1A<3 might be more favorable for controlling high blood pressure in hypertensive patients. Moreover, regarding the in vitro assays, our results suggest that the antihypertensive effect of RBPH-1A<3 could be related to their potent ACE inhibitory and antioxidant activity, while RBPH-2F<3 only showed antioxidant properties. Nevertheless, further studies are needed to determine whether other mechanisms could also be established regarding the blood-pressure-lowering effect of both hydrolysates.

**Figure 3:**
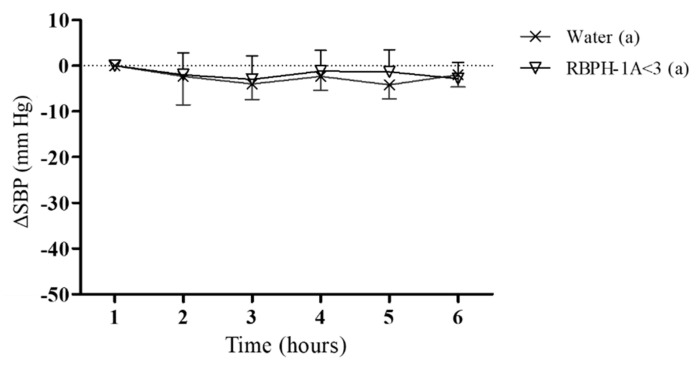
Decrease in the systolic blood pressure caused in Wistar–Kyoto (WKY) rats after the administration of water (x) or 80 mg/kg of the rice bran protein hydrolysate fraction with molecular mass smaller than 3 kDa RBPH-1A<3 (▽). Data are expressed as mean ± SEM. The experimental groups always had a minimum of eight animals. No statistical differences were observed. The same letters indicate no statistical differences (*p* > 0.05). Significance was estimated using two-way ANOVA.

To evaluate whether the blood-pressure-lowering effect produced by RBPH-1A<3 was dependent on the hypertensive condition, we also evaluated the effect of this hydrolysate in normotensive WKY rats (Figure 3). These animals are frequently used like the normotensive control of the SHR strain because they present the same origin and genetic charge [50].

The administration of 80 mg/kg RBPH-1A<3 did not modify the SBP in normotensive animals when compared to the control group, suggesting that this hydrolysate would not exert an hypotensive effect in normotensive subjects.

In this study, we have demonstrated that hydrolysates from rice bran proteins possess potent in vitro antioxidant and ACE inhibitory properties, as well as in vivo activity, to decrease arterial blood pressure in hypertensive rats. These properties can be attributed to the presence of peptides with a low molecular mass, in addition to the phenolic compounds released during hydrolysis processes. These findings provide new evidence of the potential of the industrial by-product of rice milling to be used as a source of bioactive compounds that can improve cardiovascular health and alleviate other related diseases. It is important to note that the potential peptide sequences responsible for the antihypertensive effect have not been identified yet, but, although the majority of studies conducted in recent years have focused on the isolation of peptide sequences released during hydrolysis, it has recently been shown that the administration of complete hydrolysates could be more relevant than the administration of a single isolated peptide, since a greater biological effect could be achieved. Although we consider that hydrolysates could be more interesting products for the development of functional foods from a technological and organoleptic point of view [51], further investigation is needed to identify the potential sequences responsible for the bioactivity and the antihypertensive mechanisms and pathways implicated in the effect produced by these hydrolysates.

## Figures and Tables

**Table 1:** ORAC (oxygen radical absorbance capacity) values and reducing power analysis of defatted rice bran protein concentrates (DRBPC-1 and DRBPC-2), protein hydrolysates with alcalase (RBPH-1A and RBPH-2A) and flavorzyme (RBPH-1F and RBPH-2F), and fractions with molecular mass smaller than 3 kDa (RBPH-1A<3, RBPH-2A<3, RBPH-1F<3, and RBPH-2F<3). Values given are the means ± standard deviation of three measurements. The same letters indicate no statistical differences (*p* > 0.05). Significance was estimated using one-way ANOVA.

Sample	ORAC (µmol eq Trolox/g Sample)	Reducing Power ABS (700 nm)
DRBPC-1	111.15 ± 5.30 ^g^	0.32 ± 0.03 ^c^
DRBPC-2	256.50 ± 10.26 ^f^	0.55 ± 0.02 ^b^
RBPH-1A	385.81 ± 12.02 ^d,e^	0.33 ± 0.02 ^c^
RBPH-2A	508.02 ± 14.42 ^d^	0.51 ± 0.03 ^b^
RBPH-1F	273.88 ± 13.99 ^e,f^	0.33 ± 0.02 ^c^
RBPH-2F	391.68 ± 53.36 ^d,e^	0.53 ± 0.03 ^b^
RBPH-1A<3	886.76 ± 32.13 ^b^	0.47 ± 0.01 ^b^
RBPH-2A<3	1029.03 ± 83.72 ^a^	0.53 ± 0.01 ^b^
RBPH-1F<3	720.03 ± 20.06 ^c^	0.96 ± 0.07 ^a^
RBPH-2F<3	1040.27 ± 73.16 ^a^	0.88 ± 0.01 ^a^

ABS = absorbance.

**Table 2:** Total phenolic compounds (mg gallic acid equivalents (GAE)/g sample) of defatted rice bran protein concentrates (DRBPC-1 and DRBPC-2), protein hydrolysates with alcalase (RBPH-1A and RBPH-2A) and flavorzyme (RBPH-1F and RBPH-2F), and fractions with molecular mass smaller than 3 kDa (RBPH-1A<3, RBPH-2A<3, RBPH-1F<3, and RBPH-2F<3). Values are given as the means ± standard deviation of three measurements. The same letters indicate no statistical differences (*p* > 0.05). Significance was estimated using one-way ANOVA.

Sample	mg GAE/g Sample
DRBPC-1	4.78 ± 0.63 ^e^
DRBPC-2	7.90 ± 0.52 ^d,e^
RBPH-1A	15.81 ± 0.65 ^c^
RBPH-2A	19.38 ± 1.33 ^c^
RBPH-1F	9.43 ± 0.87 ^d^
RBPH-2F	15.50 ± 1.75 ^c^
RBPH-1A<3	36.17 ± 1.90 ^a^
RBPH-2A<3	37.24 ± 2.58 ^a^
RBPH-1F<3	29.01 ± 1.80 ^b^
RBPH-2F<3	34.23 ± 0.77 ^a^

**Table 3:** Inhibition of the angiotensin-converting enzyme (ACE) expressed as IC_50_ presented by the fractions with molecular mass smaller than 3 kDa from defatted rice bran protein hydrolysates (RBPH-1A<3, RBPH-2A<3, RBPH-1F<3, and RBPH-2F<3). The values are given as the means ± standard deviation of three measurements. The same letters indicate no statistical differences (*p* > 0.05). Significance was estimated using one-way ANOVA.

Sample	IC_50_
	(mg Sample/mL)
RBPH-1A<3	0.15 ± 0.02 ^d^
RBPH-2A<3	0.32 ± 0.09 ^c^
RBPH-1F<3	1.70 ± 0.16 ^a^
RBPH-2F<3	1.42 ± 0.06 ^b^
